# A List of the Alumni of the Ohio College of Dental Surgery, from Its Organization to the Present Time

**Published:** 1859-11

**Authors:** 


### A LIST OF THE ALUMNI

**Of the Ohio College of Dental Surgery, from its organization to the present time.**

At the suggestion of various persons, we have concluded to publish a list of the graduates of the Ohio Dental College. Inquiries have been made frequently as to who the early graduates were; to answer those inquiries, and in justice to the alumni, we give their names and present location, so far as we can, also the date of their graduation. If there are any inaccuracies, we shall be pleased to be notified of the same, and correction will be made.—EDS.

CLASS OF 1846.

| | |
|---|---|
| *W. B. Ross, | |
| B. A. Satterthwaite, | Dayton, Ohio. |
| *D. P. Hunt, | |
| A. Berry, | Raymond, Miss. |

CLASS OF 1847.

| | |
|---|---|
| H. R. Smith, | Cincinnati, O. |
| A. M. Leslie, | St. Louis, Mo. |
| G. L. Van Emon, | Nashville, Tenn. |
| P. Knowlton, | Cincinnati, O. |
| A. S. Talbert, | Lexington, Ky. |

CLASS OF 1848.

| | |
|---|---|
| J. S. King, | Pittsburgh, Pa. |
| Samuel Griffith, | Louisville, Ky. |
| J. A Weaver, | Batavia, O. |
| *B. J. Dudley, | |
| C. F. Knowlton, | Detroit, Mich. |
| Y. W. Lewis, | Howell, Mich. |
| W. H. Goddard, | Louisville, Ky. |

CLASS OF 1849.

| | |
|---|---|
| E. Bray, | Evansville, La. |
| *A. L. Dwyer, | |
| T. R. Willard, | Dayton, Ohio. |
| Wm. S. Chandler, | New Orleans, La. |
| Amos Pratt, | Henry, Ills. |
| H. D. Stratton, | St. Louis, Mo. |
| S. B. Fithian, | " " |
| J. H. Andrews, | Yazoo, Miss. |

* Deceased.

### CLASS OF 1850.

| | |
|---|---|
| J. Ramsay, | Springfield, O. |
| Wm. H. King, | Lancaster, O. |
| J. Taft, | Cincinnati, O. |
| J. W. Keys, | Florida. |
| H. E. Peebles, | St. Louis, Mo. |
| Wm. Bell. | Elizaville, Ky. |

### CLASS OF 1851.

| | |
|---|---|
| E. Buckwell, | California. |
| John G. Hamill, | Lancaster, O. |
| J. C. Weaver, | Batavia, O. |
| J. C. Clark, | Circleville, O. |

### CLASS OF 1852.

| | |
|---|---|
| W. S. Burke, | Porto Rico. |
| W. C. Duncan, | Cincinnati, O. |
| G. L. Payne, | Xenia, O. |
| J. C. Whinnery, | Salem, O. |
| J. A. Herring, | Kosciusko, Miss. |
| A. L. Dwyer, | Greenfield, O. |
| M. N. Manlove, | Lafayette, Ind. |
| J. L. Knapp, | New Orleans, La. |
| Samuel P. Cutter, | Holly Springs, Miss. |
| D. Dougherty, | Bardstown, Ky. |
| C. W. Spalding, | St. Louis, Mo. |
| A. D. Bigelow, | Cleveland, O. |

### CLASS OF 1853.

| | |
|---|---|
| J. T. Irwin, | Cincinnati, O. |
| J. C. Ross, | Nashville, Tenn. |
| John Harris, | Salem, O. |
| J. L. Milton, | Yazoo City, Miss. |
| H. A. McDaniel, | Nashville, Tenn. |
| H. E. Eames, | Lebanon, Tenn. |
| J. M. Lewis, | Quincy, Ills. |

| | |
|---|---|
| J. F. Johnson, | Indianapolis, Ind. |
| Joseph Richardson, | Cincinnati, O. |
| C. H. Quinlan, | Chicago, Ills. |
| E. S. Holmes, | |
| G. W. Keely, | Oxford, O. |
| S. D. Muse, | Sharon, Miss. |
| J. A. Kennicott, | Chicago, Ills. |

CLASS OF 1854.

| | |
|---|---|
| G. Watt, | Xenia, O. |
| E. Collins, | Connersville, Ind. |
| *B. N. Freeman, | |
| M. W. S. Kendall, | Illinois. |
| E. A. Hermon, | Nashville, Tenn. |
| A. L. Brown, | London, O. |
| G. Laurie, | London, England. |
| M. DeCamp, | Mansfield, O. |
| J. W. Baxter, | Warsaw, Ky. |
| P. G. C. Hunt, | Indianapolis, Ind. |

CLASS OF 1855.

| | |
|---|---|
| J. E. Jones, | Dayton, O. |
| H. Munro, | Lebanon, Ky. |
| W. A. Cornelius, | |
| J. Douthett, | Xenia, O. |
| R. C. Reid, | Anderson, Ind. |
| *T. L. Waring, | |
| G. B. Miner, | Milwaukee, Wis. |
| J. D. Quinlan, | Chicago, Ills. |
| *A. J. Reeve, | |
| H. B. McKellops, | St. Louis, Mo. |
| J. G. Fredericks, | New Orleans, La. |
| J. B. Newbrough, | |

CLASS OF 1856.

| | |
|---|---|
| A. D. Nevins, | Lexington, Miss. |
| E. Osmond, | Cincinnati, O. |

* Deceased.

| | |
|---|---|
| J. R. James, | Volney, Ky. |
| F. Bell, | Cincinnati, O. |
| T. Davenport, | " " |
| C. H. James, | " " |
| F. Grimes, | Fayette, Mo. |
| J. F. Mercer, | Canada West. |
| W. G. Drake, | Aberdeen, Miss. |
| A. G. Stipher, | Kentucky. |

CLASS OF 1857.

| | |
|---|---|
| G. W. Hall, | |
| H. A. Smith, | Cincinnati, O. |
| Chas. J. May, | Illinois. |
| Chas. Silsbee, | |
| A. G. Phillips, | Ravenswood, Va. |

CLASS OF 1858.

| | |
|---|---|
| C. C. Dills, | Peru, Iowa. |
| M. W. Wortman, | Hamilton, C. W. |
| W. A. Ross, | Covington, Ky. |
| S. L. Bracy, | Raymond, Miss. |
| C. W. Robinson, | Greenfield, O. |

CLASS OF 1859.

| | |
|---|---|
| L. B. Hatch, | Lebanon, Ky. |
| J. B. Doughty, | Mississippi. |
| J. Ingalls, | Wisconsin. |
| J. B. Knisely, | Decatur, Ills. |
| G. B. Lenoir, | Monticello, Miss. |
| W. B. Ward, | Camden, Ark. |
| E. Ruth, | Covington, Ky. |
| O. Willson, | Aurora, Ills. |
| S. Wardell, | Cincinnati, O. |
| W. H. Atkinson, | Cleveland, O. |
| W. W. Allport, | Chicago, Ills. |
| E. Taylor, | Palmyra, Mo. |

